# Metformin prevents cancer metastasis by inhibiting M2-like polarization of tumor associated macrophages

**DOI:** 10.18632/oncotarget.5541

**Published:** 2015-10-19

**Authors:** Ling Ding, Guikai Liang, Zhangting Yao, Jieqiong Zhang, Ruiyang Liu, Huihui Chen, Yulu Zhou, Honghai Wu, Bo Yang, Qiaojun He

**Affiliations:** ^1^ Zhejiang Province Key Laboratory of Anti-Cancer Drug Research, Institute of Pharmacology and Toxicology, College of Pharmaceutical Sciences, Zhejiang University, Hangzhou 310058, China

**Keywords:** metformin, macrophage polarization, cancer metastasis, AMPKα1

## Abstract

Accumulated evidence suggests that M2-like polarized tumor associated macrophages (TAMs) plays an important role in cancer progression and metastasis, establishing TAMs, especially M2-like TAMs as an appealing target for therapy intervention. Here we found that metformin significantly suppressed IL-13 induced M2-like polarization of macrophages, as illustrated by reduced expression of CD206, down-regulation of M2 marker mRNAs, and inhibition of M2-like macrophages promoted migration of cancer cells and endothelial cells. Metformin triggered AMPKα1 activation in macrophage and silencing of AMPKα1 partially abrogated the inhibitory effect of metformin in IL-13 induced M2-like polarization. Administration of AICAR, another activator of AMPK, also blocked the M2-like polarization of macrophages. Metformin greatly reduced the number of metastases of Lewis lung cancer without affecting tumor growth. In tumor tissues, the percentage of M2-like macrophage was decreased and the area of pericyte-coated vessels was increased. Further, the anti-metastatic effect of metformin was abolished when the animals were treated with macrophages eliminating agent clodronate liposome. These findings suggest that metformin is able to block the M2-like polarization of macrophages partially through AMPKα1, which plays an important role in metformin inhibited metastasis of Lewis lung cancer.

## INTRODUCTION

Macrophages are a major cellular component of murine and human tumors, where they are commonly termed tumor-associated macrophages (TAMs). Although the original hypotheses proposed that TAMs are involved in anti-tumor immunity, recent clinical and experimental evidences show tumor-promoting effect of TAMs in various cancers [1–4]. Epidemiological studies indicate a strong association between increased macrophage infiltration and poor prognosis and in ovarian, cervical, thyroid, lung, hepatocellular cancers, and breast cancers [5–9]. Analysis of the transcriptome of TAMs derived from mouse models of breast cancer has also provided evidence that enrichment in macrophage transcripts is predictive of poor prognosis and reduced survival in human breast cancer [10, 11]. Specific depletion of macrophages using clodronate-encapsulated liposomes reduces growth in melanoma, ovarian, Lewis lung, teratocarcinoma, rhabdomyosarcoma, and prostate tumor graft models [6, 12–14].

Because plasticity and flexibility are key features of macrophages and their activation sates, whether TAMs execute tumor-preventing or tumor-promoting role depends on their polarization statues [15, 16]. Macrophage activation is broadly categorized as classically activated, or M1, and alternatively activated, or M2. In nonmalignant or regressing tumors, the majority of TAMs is classic activated macrophages (M1-like), representing pro-inflammatory activity, presenting antigen and promoting tumor lysis. On the contrary, TAMs in malignant tumors tend to resemble alternatively activated macrophages (M2-like), which enhance tumor-associated angiogenesis, promote the ability of tumor migration and invasion, as well as suppress the antitumor immune responses. Thus, M2-like TAMs are considered to be a potential targets for adjuvant anticancer therapies and recent therapeutic approaches targeting M2-like TAM have gained encouraging results. For example, targeted delivery of peptide to M2-like TAM improves survival of tumor bearing mouse [17]. Inhibition of CSF-1 receptor, which is essential for macrophage differentiation, significantly increased survival and suppressed established tumors, accompanied by decreased M2-like TAM [18]. Furthermore, by skewing TAM polarization away from the M2- to M1-like phenotype, HRG promotes antitumor immune responses and vessel normalization, decreases tumor growth and metastasis and enhances chemotherapy [4].

Metformin, used to be the anti-diabetic drug, is associated with decreasing cancer incidence or cancer-related mortality in diabetic patients in a compelling evidence [19–21]. Because of its excellent safety in diabetes patients, the clinical evaluation of metformin for its chemo-preventive and anti-neoplastic effects has bypassed the traditional phase I assessment and has directly moved forward to phase II and phase III trials in several cancers [22, 23]. Extensive studies have been carried out to declare the underlying mechanism for the beneficial role of metformin in cancer. Metformin mediated AMPK activation leads to an inhibition of mTOR signaling, a reduction in phosphorylation of its major down-stream effectors, the eukaryotic initiation factor 4E-binding proteins (4E-BPs) and ribosomal protein S6 kinase (SK6Ks), and an inhibition of global protein synthesis and proliferation in a number of different cancer cell lines [24]. Recent studies have demonstrated that metformin may also target cancer-initiating cells [25] or repress the process of epithelial to mesenchymal transition (EMT) [26]. However, the published studies which focused on inhibition of cell proliferation or induction of cell apoptosis, are not able to fully explain the beneficial effect of metformin in cancer.

Given that M2-like macrophages are greatly involved in cancer metastasis, we investigated the relevance between macrophage polarization and the antitumor effect of metformin. Here we showed that metformin, from 0.5 to 2.0 mM, efficiently skewed macrophages away from M2 polarization induced by IL-13. We also found that the metformin inhibits metastasis of Lewis lung cancer (LLC) *in vivo*, and this effect was abolished in macrophage eliminated system. Our study suggests that the inhibition of M2 polarization of TAMs may contribute to metformin reduced cancer incidence and cancer-related morality.

## RESULTS

### Metformin inhibits M2 polarization of macrophages induced by IL-13

**Figure 1:**
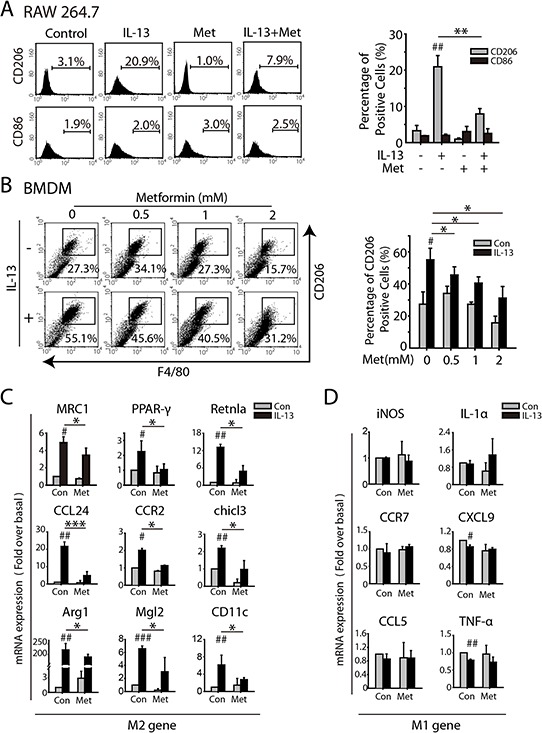
Metformin inhibits M2-like polarization of macrophages induced by IL-13 **A.** RAW264.7 were treated with IL-13(10 ng/ml), metformin (1 mM), or the combination for 72 h. Both the expression of M2 marker CD206 and M1 marker CD86 were analyzed by FACS analysis. **B.** Bone marrow-derived macrophages (BMDMs) were treated with IL-13(10 ng/ml) and different concentrations of metformin as indicated for 72 h and the percentage of F4/80^+^ CD206^+^ macrophages were determined by FACS analysis. **C.** and **D.** Quantitative RT-PCR was carried out to assess mRNA expression of M2-marker genes (C) and M1-marker genes (D) when the macrophages were treated with IL-13(10 ng/ml) for 24 h, metformin (1 mM), or both of them. The histogram bars represent three independent experiments. *p,^#^*p* < 0.05; **p,^##^*p* < 0.01; as evaluated using Student's *t* test.

RAW264.7 was exposed to serial concentrations of metformin for five days and cell growth was determined by SRB assays. As showed in Supplementary Figure S1, metformin didn't cause significant growth inhibition in the concentration from 0.5 to 4.0 mM. We first analyzed the impact of metformin on IL-13 induced M2-like polarization of macrophages. As shown in Figure 1A, significant up-regulation of CD206 were observed when RAW264.7 were treated with 10 ng/ml IL-13 for 72 h, which was greatly reduced by 1.0 mM metformin. Similarly, IL-13 induced CD206 expression in BMDMs was reduced in a concentration-dependent manner (Figure 1B). To further confirm the role of metformin in M2-like polarization, transcription changes were assessed by real-time PCR. M2 marker genes, including MRC1, PPARγ, CCL24, CCR2, chil3, Mgl2, Retnla, and Arg1, were decreased by 1 mM metformin compared with IL-13 treated group, while M1-like genes was not effected (Figure 1C and 1D). These results suggested that metformin effectively inhibited M2-like polarization of macrophage *in vitro*. Moreover, metformin inhibited PMA induced M2-like shift of THP-1 as indicated by reduced expression of CD206 and mRNA expression of MRC1 and dectin (Supplementary Figure S2).

### Metformin eliminates migration-promoting feature of M2-like macrophages

**Figure 2:**
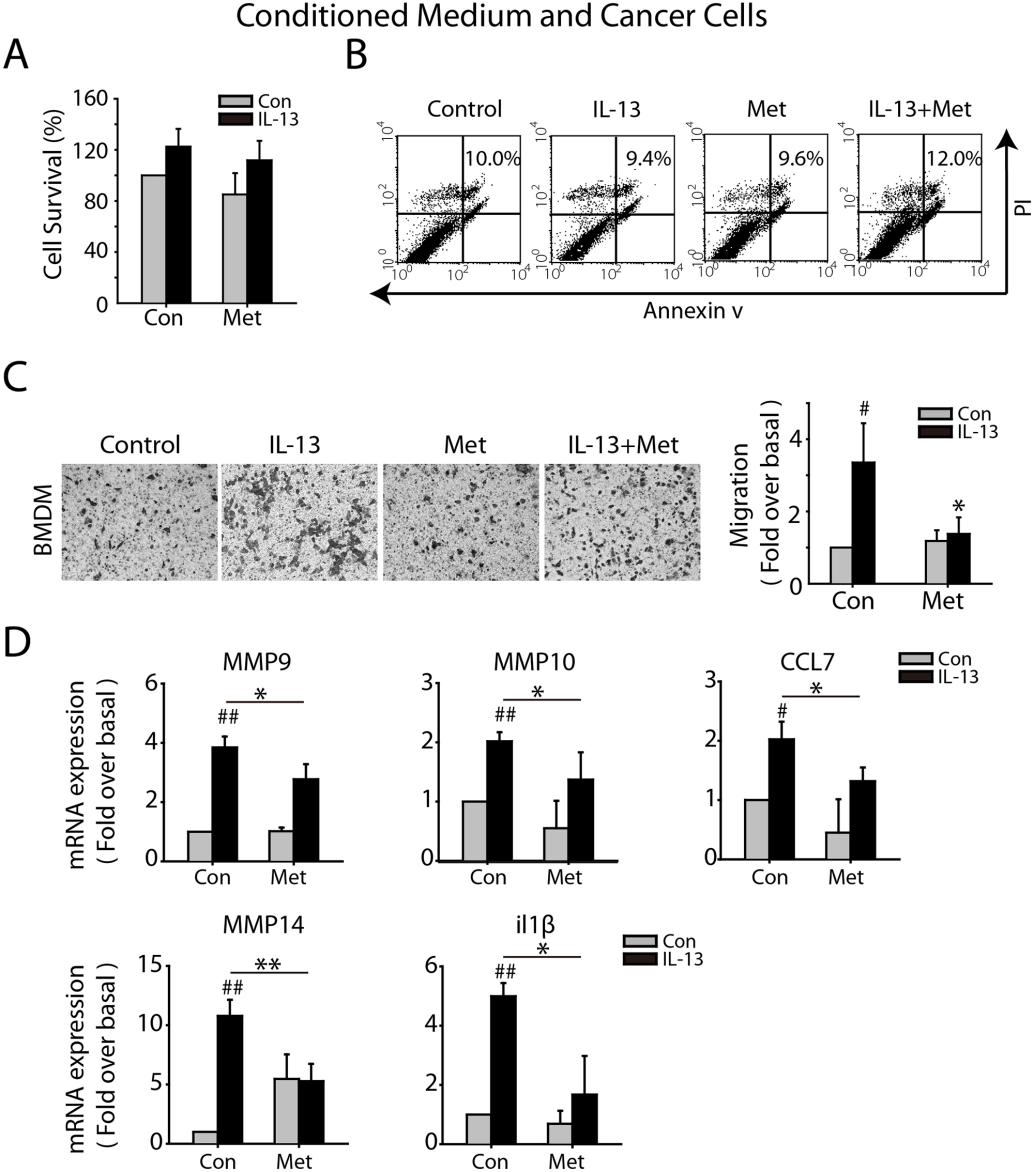
Metformin inhibits M2-like macrophages promoted migration of Lewis Cancer Cells (LLCs) *in vitro* BMDM were treated with IL-13(10 ng/ml), metformin (1 mM), or the combination for 72 h and the culture medium were replaced by fresh medium without serum, 24 h later the supernatant medium was collected as macrophage-conditioned medium (CM). **A.** LLCs were cultured with CM for 24 h and cell survival was determined by SRB assay. **B.** LLCs were cultured with CM for 24 h and cell apoptosis was determined by FACS analysis. **C.** The effect of different CMs on LLCs migration was evaluated by trans-well assay in 24 h. **D.** BMDM were treated with IL-13(10 ng/ml), metformin(1 mM), or both of them for 24 h and the mRNA expression of metastasis-relative genes were determined by quantitative RT-PCR. The histogram bars represent three independent experiments. *p, ^#^*p* < 0.05; as evaluated using Student's *t* test.

Given the capacity of M2-like macrophage to promote cancer metastasis, we investigated the impact of metformin on the functional macrophage-tumor cell interaction. Macrophages were treated with IL-13, metformin, or both of them for 72 h, and the culture medium were replaced by fresh medium without serum, 24 h later the supernatant medium was collected as CM. To exclude the impact of CM on tumor cell survival, LLC cells were treated with the conditioned medium for 24 h and cell proliferation and apoptosis were analyzed. No significant difference was found in four groups (Figure 2A and 2B). CM from IL-13 treated macrophages significantly promoted migration of LLC cells in 24 h, whereas CM from combined treatment of IL-13 and metformin didn't have this effect, nor did the CM from metformin treated macrophage (Figure 2C). Further, the expression of metastasis-related genes in macrophage with IL-13 stimulation was intensely increased, while 1 mM metformin partially blocked this increase (Figure 2D).

### Metformin inhibits angiogenesis-promoting phenotype of M2-like macrophages

**Figure 3:**
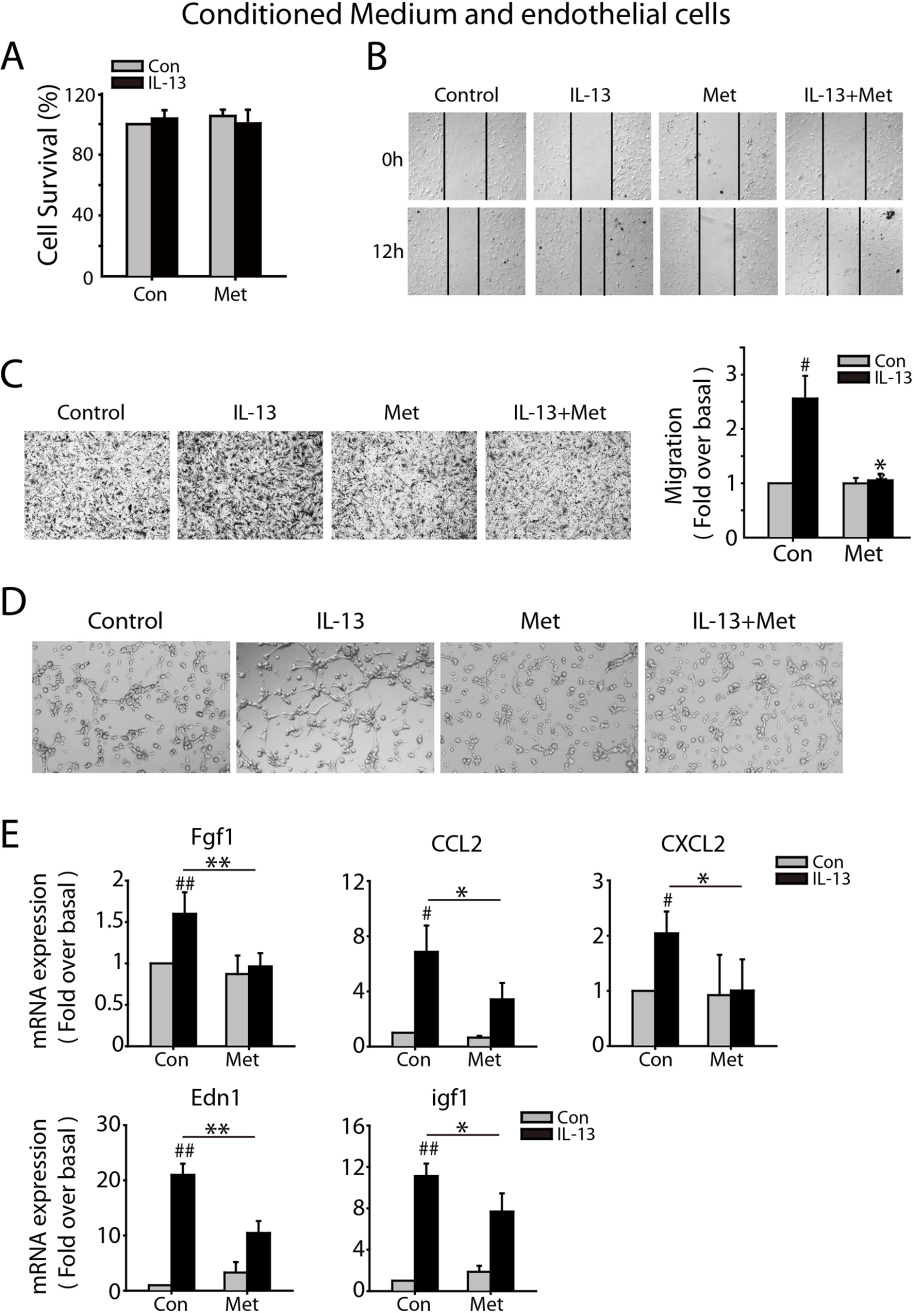
Metformin inhibits M2-like macrophages promoted angiogenesis *in vitro* **A.** CMs were collected as above. Mouse pulmonary micro-vascular endothelial cells (MMVECs) were cultured with CMs for 72 h and cell survival was determined by SRB assay. **B.** For wood-healing assay, MMVECs were scratched with a pipette tip and then treated with CMs for 12 h. **C.** Trans-well assay was carried out to evaluate the impact of CMs on migration of MMVECs in 24 h. **D.** For tube formation assay, MMVECs were seeded in 96-well plates filled with matrigel and incubated with different CMs. After 6 h, cells were photographed under phase contrast microscopy (5X). **E.** Quantitative RT-PCR was carried out to measure the mRNA expression of angiogenesis-relative genes in macrophages treated with IL-13(10 ng/ml), metformin(1 mM), or both of them for 24 h. The histogram bars represent three independent experiments. *p, ^#^*p* < 0.05; as evaluated using Student's *t* test

There is a growing appreciation that M2-like macrophages tend to be angiogenesis-promoting phenotype, therefore we analyzed the effect of metformin on the angiogenesis-promoting feature of macrophages. CM was obtained in the same way as above and we evaluated the migrating ability of MMVECs in different CM by using wound-healing assay and migration assay. The purified MMVECs were confirmed by FACs analysis by CD31staining (Supplementary Figure S3). MMVECs were treated with CM for 24 h and no significant difference in cell survival was found (Figure 3A). Both wound healing and trans-well assay revealed that CM from IL-13 challenged macrophages promoted MMVECs migration, which was abrogated by metformin (Figure 3B and 3C). Further, CM from IL-13 treated macrophage significantly promoted tube formation of MMVECs, while the CM from macrophage with combined treatment of IL-13 and metformin didn't have this effect (Figure 3D). Further, IL-13 treatment induced mRNA expression of angiogenesis-promoted genes, including Fgf1, CCL2, Edn1, CXCL2 and igf1, which was suppressed by 1 mM metformin (Figure 3E).

### AMPKα1 is involved in metformin inhibited M2-like polarization of macrophages

**Figure 4:**
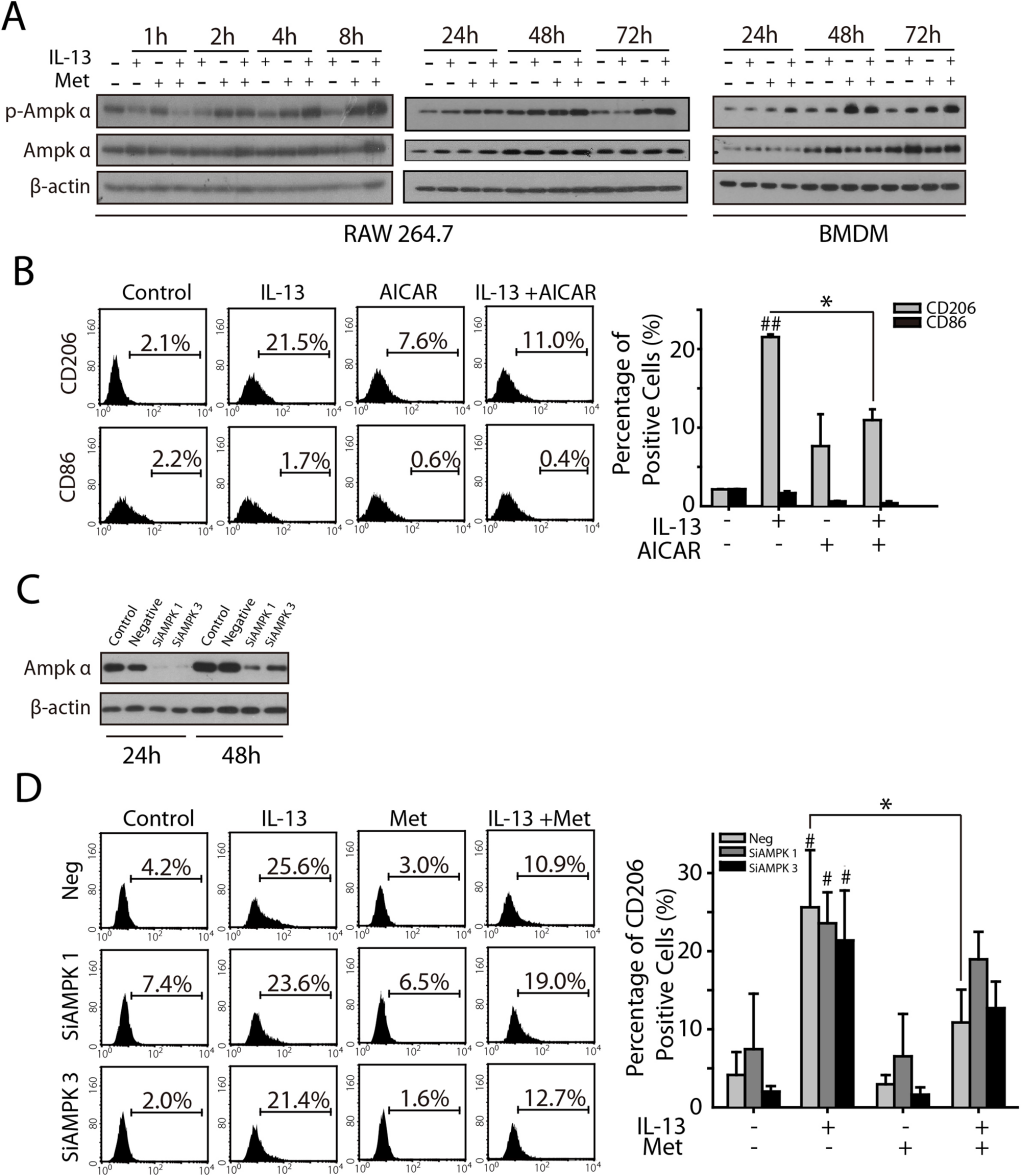
AMPKα1 is involved in metformin inhibited M2-like polarization of macrophages **A.** AMPKα1 phosphorylation in either RAW264.7 or BMDMs was determined by Western-blotting when treated with merformin (1 mM) for indicated times. **B.** RAW264.7 was treated with IL-13 (10 ng/ml), AICAR (1 mM), or both of them for 72 h, the expression of CD206 or CD86 were determined by FACS analysis. **C.** AMPKα1 in RAW264.7 was knocked down by RNA interference. **D.** The percentage of CD206^+^ macrophages was determined by FACS analysis when RAW264.7 was treated with IL-13(10 ng/ml), metformin(1 mM), or both of them in the absence of AMPKα1. All experiments were repeated at least three times. *p,^#^*p* < 0.05; **p,^##^*p* < 0.01; as evaluated using Student's *t* test.

Considering that metformin was well known for its function in activating AMPKα1, we assessed whether AMPKα1 played an important role in metformin inhibited M2-like polarization of macrophages. Since AMPKα1 is the major subunit expressed in macrophages, we analyzed the phosphorylation level of AMPKα1 during the polarization process with or without metformin. In RAW264.7 and BMDMs, IL-13 treatment didn't affect AMPKα1 or phosphorylated AMPKα1 in 1, 2, 4, 8, 24, 48, and 72 h. Whereas, metformin triggered a significant AMPKα1 phosphorylation in 4 h, which remained elevated in 24, 48, and 72 h (Figure 4A). Further, the effect of metformin in M2-like polarization inhibition was abolished when AMPKα1 was silenced (Figure 4C and 4D). AIRCA, another AMPK activator, was found to prevent the M2-like polarization stimulated by IL-13 as also (Figure 4B). Taken together, these results suggested that activation of AMPKα1 played an important role in metformin inhibited M2-like polarization.

### Metformin inhibits metastasis of LLC *in vivo* by targeting macrophages

**Figure 5:**
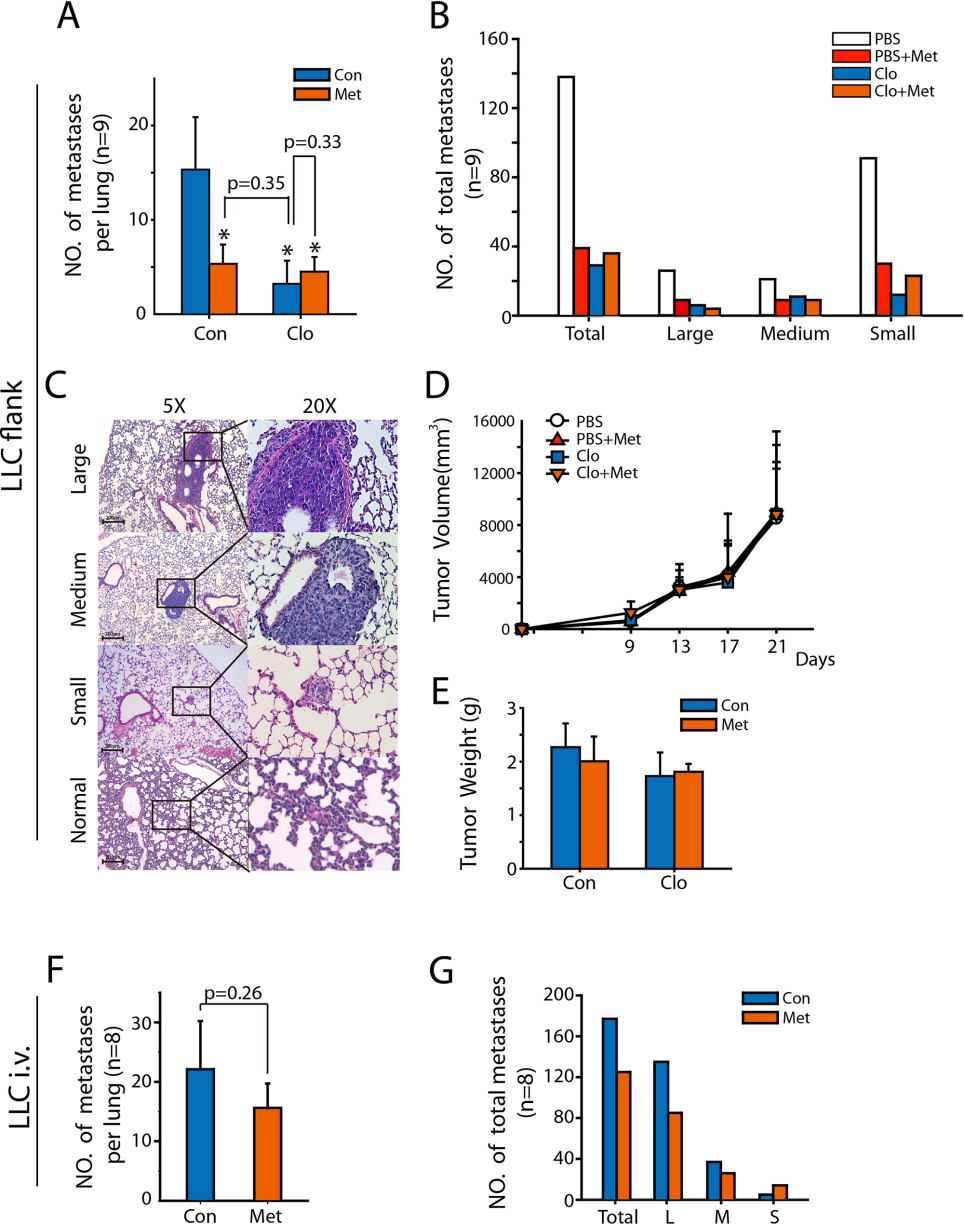
Merfromin inhibits metastasis of LLC *in vivo* by targeting macrophages **A–E.** C57BL/6 mice were injected subcutaneously with LLCs in the flank and were treated with metformin (100 mg/kg), clodronate lipsome, or both of them. After 21days, animals were sacrificed and lungs were histologically analyzed for the occurrence of metastases. Total number as well as the number of small (diameter: < 50 μm), medium-sized (diameter: 50–200 μm), or large (diameter: > 200 μm) lung metastases determined (*n* = 8, A and B). The images show representative stained lung sections (C) Tumor volume and tumor weight were determined (D and E). **F, G.** C57BL/6 mice were injected intravenously with LLCs and treated with metformin for 21 days. Total number as well as the number of small, medium-sized, or large lung metastases determined (*n* = 8, F and G).

To evaluate the impact of metformin *in vivo*, we analyzed the impact of metformin in tumor growth and metastasis of LLC. While the growth of primary tumors after subcutaneous injection of LLC was indistinguishable between control and metformin treated mice (Figure 5D and 5E), the number of metastasis was strongly reduced in metformin group (Figure 5A and 5B). Further, the number of metastasis was not affected by metformin when LLC cells were injected intravenously (i.v.) (Figure 5F and 5G), suggesting the decreased metastasis was attributable to reduced escape from the primary tumor.

To investigate whether TAM played an important role in metformin inhibited tumor metastasis, we treated the tumor-bearing mice with clodronate liposome to eliminate TAMs chemically (Supplementary Figure S4A). Consistent with previous studies, lung metastasis of LLC was greatly reduced in the depletion of TAMs and treatment of metformin did not further affect metastasis under the elimination of TAMs (Figure 5A and 5B). In addition, either treatment with metformin or clodronate liposome had no effect in tumor volume, tumor weight, and body weight (Figure 5D, 5E, and Supplementary Figure S4B). Representative HE staining of metastasis nude in lung was shown in Figure 5C.

**Figure 6:**
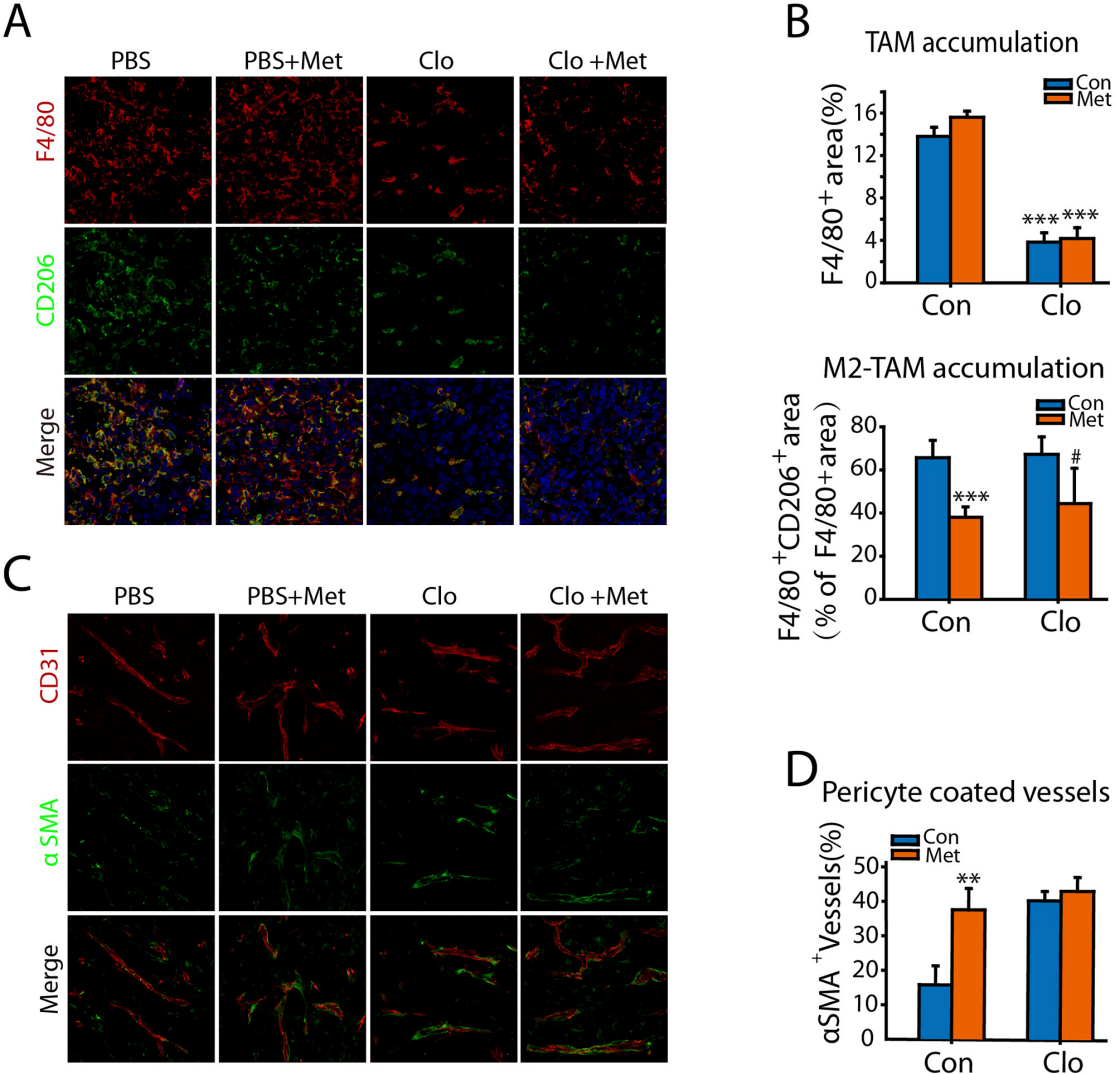
Metformin suppresses M2 polarization of TAMs and promotes tumor vessel maturation **A.** The sections of tumor tissues from different group were double stained with macrophage marker F4/80 and M2-marker CD206. **B.** F4/80^+^ area (% of tumor area) and the F4/80^+^CD206^+^ area (% of F4/80^+^ tumor area) were qualified. **C.** The sections of tumor tissues from different group were double stained with CD31 and α-SMA. **D.** α-SMA^+^ CD31^+^ area (% of CD31^+^ tumor area) was qualified. ^#^*p* < 0.05; **p < 0.01; ***p < 0.001; as evaluated using Student's *t* test.

We then investigated if metformin altered TAM polarization in tumor tissues. Compared to control, no significant difference in F4/80^+^ TAMs accumulation was observed in tumors treated with metformin. Whereas, metformin affect TAM polarization, as less F4/80^+^ TAMs expressed CD206 in metformin treated tumors (Figure 6A and 6B).

Since the status of tumor vessels contributes to tumor metastasis, we also analyzed the impact of metformin in vessels density and maturation. CD31 staining revealed that tumor vessel density and vessel area were comparable in tumors treated with or without metformin. Coverage of endothelial cells by mural cells renders vessels more mature, tight, and stable and reduces tumor cell intravasation. Double staining of CD31 and the mural marker α-smooth muscle actin (αSMA) revealed that more αSMA^+^ covered tumor vessels in tumor tissue from metformin treated mice (Figure 6C and 6D).

## DISCUSSION

In recent studies, it is reported that metformin could block the invasion and metastasis in several types of cancers, including endometrial carcinoma, ovarian cancer, melanoma and breast cancer. Huang YP et al reported that metformin blocked migration and invasion of tumor cells by inhibition of matrix metalloproteinase-9 (MMP-9) [29]. Michael C et al. reported that the inhibition of cell invasion by metformin was correlated with modulation of expression of proteins involved in epithelial mesenchymal transition such as Slug, Snail, SPARC, fibronectin and N-Cadherin [30]. Several studies also showed that metformin is able to inhibit angiogenesis in different model. Katiuscia D et al. reported that metformin may inhibit angiogenesis *in vitro* and *in vivo* by directly targeting endothelial cells [31]. A very recent study showed that metformin inhibited angiogenesis and metastatic growth of breast cancer *in vivo* [32]. However, the mechanisms of action by which metformin inhibits cancer invasion and metastasis are not fully understood. Here, we found that metformin efficiently suppressed the metastasis of LLCs *in vivo*, while this effect was abolished in macrophage depletion model. In Algire C's study, subcutaneous LLC model was used to analyze the inhibitory effect of metformin on tumor growth. They observed that metformin treatment significantly decrease tumor growth rate and tumor size in mice on the high-energy diet. However, there was no statistically significant effect of metformin on tumor growth in those mice on the control diet [33]. Similarly, we also didn't find significant inhibitory effect of metformin on tumor growth. Since a large number of evidence has proved that macrophages in the micro-environment of cancer, especially M2-like TAMs, stimulate angiogenesis, enhance tumor cell migration and invasion, our results established the relevance between macrophage and the anti-metastasis effect of metformin, suggesting that the anti-metastasis effect of metformin could at least be partially attributed to its role in the inhibition of TAM polarization.

In the present study we showed for the first time that metformin could skew macrophages away from M2 polarization *in vivo* and *in vitro*, which contributed to metformin inhibited lung metastasis of LLC cells. Further, we also demonstrated that AMPKα1 was involved in metformin prevented M2-polarization of macrophages. This finding provides a new sight in the understanding the benefit of metformin in cancer therapy.

Several studies have reported the impact of metformin on biological functions of macrophages. Metformin inhibited IL-1 induced release of the pro-inflammatory cytokines IL-6 and IL-8 in macrophages [34]. Metformin inhibits HMGB1 release in LPS-treated RAW264.7 cells and increases survival rate of endotoxaemic mice [35]. A very recent study showed that metformin exhibits anti-inflammatory action in LPS- activated macrophages via ATF-3 induction [36]. By the employment of IL-13 induced M2 polarization model, we found that metformin was able to inhibit IL-13 induced expression of M2 marker (CD206) and M2 marker mRNA (MRC1, Arg1, Retnla). Considering that in most case, the patterns of gene expression of macrophages in response to various stimuli, are heterogeneous and do not precisely fit the published patterns associated with these M1/M2 designations, we further analyzed the function phenotype of macrophages. Consistent with previous studies, our data showed that the condition medium from IL-13-activated macrophages promoted migration of LLC cells and MMVECs, and inhibited the tube formation of MMVECs. Co-treatment with metformin eliminated these tumor-promoting phenotype induced by IL-13. It should be noted that in our system metformin didn't affect the migration of LLC cells or MMVEC directly. These results indicated that M2-like phenotype of macrophage induced by IL-13 was blocked by metformin.

The effect of AMPKα in macrophage polarization has been reported in two studies. In the study of Sag et al, they found that stimulation of macrophages with IL-10 resulted in the rapid phosphorylation of AMPKα, whereas stimulation of macrophages with LPS resulted in AMPKα de-phosphorylation [37]. Further, they showed that expression of dominant negative AMPKα enhanced LPS-induced TNFα and IL-6. In contrast, transfection with a constitutively active form of AMPKα reduced LPS-induced TNFα and IL-6. However, they didn't further analyze the role AMPKα in IL-10 induced polarization of macrophages. Although IL-10 is considered to be an inducer for alternative polarization of macrophages, the signaling pathways and inflammatory response to IL-10 stimulation are greatly different from that for IL-4 and IL-13 stimulation [38–40]. In our study, we didn't found the activation of AMPKα in either RAW264.7 or BMDMs. The silence of AMPKα didn't affect IL-13 induced CD206 expression. This data suggested that AMPKα might not be required for IL-13 induced polarization.

In the study of Mounier et al, they found that BMDM from AMPKα−/− mouse showed reduced expression of CD206 and CD163 in stimulation of IL-4 or IL-10, whereas the expression of Arg1 was not affected [41]. They also didn't find significant difference of marker mRNA expression between wild type and AMPKα−/− macrophages. In our study, we found both metformin and AICAR significantly inhibited IL-13 induced expression of CD206. Further, the silence of AMPKα didn't cause significant change in IL-13 induced CD206 expression, while greatly abolished the effect of metformin inhibited CD206 expression. Our result suggests that AMPKα is at least partially responsible for the metformin.

The anticancer effects of metformin are associated with both direct (insulin-independent) and indirect (insulin-dependent) actions of the drug. The indirect, insulin-dependent effects of metformin are mediated by the ability of AMPK to reduce blood insulin, which plays a major role in its anticancer activity since insulin has mitogenic and pro-survival effects. The direct, insulin-independent effects of metformin originate from LKB1-mediated activation of AMPK and a reduction in mTOR signaling and protein synthesis in cancer cells. Some recent reports raise the possibility that metformin may mediate additional anticancer effects independently of AMPK, LKB1, and TSC2. Unlike these previous studies, our data suggested that metformin may target angiogenesis in an indirect way by inhibiting M2 polarization of macrophage.

In summary, we report the first evidence that metformin inhibits M2-like polarization of macrophages both *in vitro* and *in vivo*, which contributes to metastasis prevention role of metformin. Our finding suggests that in addition to lower circulating insulin and direct inhibitory effects on cancer cells, the TAMs by is a potential new in the understanding the benefit in cancer therapy. Our findings link TAM to the anti-metastatic effect of metformin, providing further support for clinical application of metformin.

## MATERIALS AND METHODS

### Ethics statement

Investigation has been conducted in accordance with the ethical standards and according to the Declaration of Helsinki and according to national and international guidelines and has been approved by the authors' institutional review board.

### Reagents

Metformin, LPS, AICAR, and phorbol 12-myristate 13-acetate (PMA) were obtained from Sigma (St. Louis, MO). Recombinant murine IL-13 was purchased from PeproTech (Rocky Hill, NJ). Mouse recombinant M-CSF and antibodies against AMPKα1 and p-AMPKα1 were from Cell Signaling Technology (Beverly, MA, USA). Antibody against Actin was purchased from Santa Cruz Biotechnology (CA, USA). Antibodies for flow cytometry including PE-conjugated anti-mouse CD206, PE-conjugated anti-mouse CD86 and FITC-conjugated anti-mouse F4/80 were purchased from Biolegend (San Diego, CA, USA). For immunofluorescence, first antibodies including anti-mouse F4/80, anti-CD31, anti-αSMA, and FITC-conjugated anti-mouse CD206 were from eBioscience, Abcam, Sigma and Biolegend respectively, while secondary antibodies including anti-Rat and anti-mouse were from life technology. FITC Annexin V Apoptosis Detection Kit I and matrigel were purchased from BD (San Jose, CA, USA). JetPrime transfection agent was obtained from Polyplus. Clodronate liposomes and PBS liposomes were purchased from ClodronateLiposomes.com (Amsterdam, Netherlands).

### Cell culture and differentiation

Lewis Lung cancer (LLC) cells, RAW 264.7, and THP-1 were obtained from the Cell Bank of the China Science Academy (Shanghai, China). RAW 264.7 and LLC cells were cultured in DMEM, THP-1 was maintained in RPMI-1640 medium, contained 10% FBS and 100 U per ml of penicillin-streptomycin in a 5% CO_2_ humidified incubator at 37°C. THP-1 was seeded in 6-well plates in a density of 2 × 10^5^/well and differentiated by 200 nM PMA for 48 h.

### Tumor models and macrophage depletion

C57BL/6 (4–5 weeks old) mice were obtained from National Rodent Laboratory Animal Resource (Shanghai, China). LLCs subcutaneous model: 1 × 10^6^ cells in 0.2 ml DMEM were injected subcutaneously to the flanks of C57BL/6. Metformin was given at 100 mg/kg via intraperitoneal injection 12 h before tumor-cell injection, and once a day thereafter for prolonged treatments. There were 9 mice in each group. Animals were sacrificed 21 days after injection of tumor cells, and tumors were weighed.

LLCs intravenous model: C57BL/6 mice were injected intravenously with 1 × 10^5^ cells in 0.1 ml DMEM. Metformin was given at 100 mg/kg via intraperitoneal injection 12 h before tumor-cell injection, and once a day thereafter for prolonged treatments. There were 8 mice in each group. Animals were sacrificed 21 days after injection of tumor cells.

Macrophage depletion was achieved by intraperitoneal injection of a loading dose of 0.1 ml/10 g of the liposome suspension, followed by repeated injections of 0.05 ml/10 g every fourth day to prevent repopulation of macrophages. The efficiency of macrophage depletion was assessed by immunostaining of liver, spleen, and tumor sections for F4/80.

For analysis of pulmonary metastases, lungs were removed and fixed in paraformaldehyde, cut in 3 μm sections, and stained for hematoxylin and eosin. One section every 200 μm throughout the whole lung was screened histologically, and the number of metastases was counted and assigned to the respective size category: small (diameter: <50 μm), medium (diameter: 50–200 μm) or large (diameter: >200 μm).

### Immunofluorescence

To analyze the expression of M2-like macrophages, tumor tissues were immediately frozen in OCT compound. For the evaluation of tumor vessel normalization, tumor tissues were fixed in 4% PFA for 1 h, dehydrated overnight at 4°C and then frozen in OCT compound. And then all the tissues were cut at 8 μm thickness. For immunofluorescence, following primary antibodies were used: rat anti-F4/80(1:200), rat anti-CD31(1:200), mouse anti-alpha smooth muscle actin(1:200). Then approximate secondary fluorescent antibodies conjugated with Alexa Fluor 488 or 594 were incubated. For the analysis of M2-like macrophage, FITC-conjugated anti-mouse CD206 antibody (1:200) was incubated for 4 h at room temperature. Then nuclei were visualized by staining DAPI for 5 min. For morphometric evaluation, at least five optical fields per tumor section were randomly chosen, analyzed by Olympus IX81-FV1000 confocal laser-scanning microscope. For all the studies, 5–10 optical fields (20× or 40× magnification) per tumor section were randomly chosen and analyzed. Vessel area and mural cell coverage was quantified by morphometric analysis as described.

### Bone marrow derived macrophages isolation and differentiation

Bone marrow derived macrophages (BMDMs) were produced as previously described [27] with small modification. Simply, Six week old C57BL/6 mice were sacrificed and soaked in 75% ethanol. Bone marrow cells were cultured in DMEM containing 10% FBS and 50 ng/ml M-CSF for three days to obtain BMDMs.

### Mouse pulmonary micro-vascular endothelial cells isolation

Six week-old C57BL/6 mice were sacrificed and lungs were removed and washed with PBS, then minced into small pieces and incubated with 0.1% collagenase I in 37°C for 30 min. The tissue suspension was passed through a 70 μm cell strainer and centrifuged at 1000 rpm for 5 minutes. Cells were resuspended in endothelial cell growth media (Lonza, Walkersville, ML, USA) and cultured in T75 flask. After 48 h, mouse pulmonary micro-vascular endothelial cells (MMVECs) were selected with anti-CD31-conjugated magnetic beads as previously described and the purity of MMVEC was confirmed by flow cytometry (Supplementary Figure S3).

### Cell survival assay

For the analysis of cell proliferation, cells were stained by sulforhodamine B as described previously and evaluated by the multiscan spectrum. The inhibition rate of cell proliferation for each well was calculated.

For the evaluation of cell apoptosis, cells were stained by PI/Annexin V as recommended by the manufacturer and analyzed by the BD FACS-Calibur cytometer (Becton Dickin-son, San Jose, CA).

### Flow cytometry

RAW264.7 and BMDMs were collected with scraper and blocked with 3% BSA for 45 mins, and then were incubated with PE-conjugated anti-mouse CD86 (1:100), PE-conjugated anti-mouse CD206 (1:100) antibody or FITC-conjugated anti-mouse F4/80 (1:200), according to the manufacturers' instructions. For each sample at least 1 × 10^4^ cells should be analyzed using the BD FACS-Calibur cytometer (Becton Dickin-son, San Jose, CA).

### Cell transfection

The siRNA sequence was duplexes produced by Genepharma, Co. (Shanghai, China). The sequences of siRNAs used were as follows, Si-AMPK1: sense: UGA CCGGACAUAAAGUGGCUGUGAATT, antisense: UUCAC AGCCACUUUAUGUCCGGUCA-TT; Si-AMPK3: sense: UCUCUUUCCUGAGGACCCAUCUUAUTT, antisense: AU-AAGAUGGGUCCUCAGGAAAGAGATT. The trans fection was performed using siRNA and jetPrime according to the manufacturer's recommendations.

### Quantitative PCR assay

Total RNA from BMDM was isolated using the Easy Pure RNA Kit (Transgen Biotech Co., Ltd), and cDNA was synthesized. The sequences of the primers used for the quantitative real time-PCR were listed in Table 1.

**Table 1:** Primers used for qRT–PCR analysis

Genes	Primer sequence (5′ → 3′)	
**MRC1**	Forward primer:	AGGGACCTGGATGGATGACA
	Reverse primer:	TGTACCGCACCCTCCATCTA
**PPAR-γ**	Forward primer:	TTCGATCCGTAGAAGCCGTG
	Reverse primer:	TTGGCCCTCTGAGATGAGGA
**CCL24**	Forward primer:	TGTCTGCAGTTGAGCCTACG
	Reverse primer:	GTTCGGGACCCTGGAGTTAG
**CCR2**	Forward primer:	CCTTCTCTTTCTGCAGGAAACTT
	Reverse primer:	ACAACTCACCAGGTATGGCTC
**chil3**	Forward primer:	CATGAGCAAGACTTGCGTGAC
	Reverse primer:	GGTCCAAACTTCCATCCTCCA
**MMP9**	Forward primer:	TCTAGGCCCAGAGGTAACCC
	Reverse primer:	AGGAAGGTGGACAAGCGATG
**Mgl2**	Forward primer:	CTCTGGTCTGAGGGAGAGGT
	Reverse primer:	CAAGGTAGAGGGGAGCAAGC
**CD11c**	Forward primer:	TTGCTTAGCAGTCTCTGGTGG
	Reverse primer:	TTCTGGGTCATAGGCTTGGC
**Retnla**	Forward primer:	CCCTGCTGGGATGACTGCTA
	Reverse primer:	TGCAAGTATCTCCACTCTGGATCT
**Arg1**	Forward primer:	AACACGGCAGTGGCTTTAAC
	Reverse primer:	GTCAGTCCCTGGCTTATGGTT
**iNOS**	Forward primer:	TTTGCTTCCATGCTAATGCGAAAG
	Reverse primer:	GCTCTGTTGAGGTCTAAAGGCTCCG
**IL1-α**	Forward primer:	AGGGGGTAAAAGGGGGAGAT
	Reverse primer:	AGCTGACTGCTCTGGGGATA
**TNF-α**	Forward primer:	AATGGCCTCCCTCTCATCAGTT
	Reverse primer:	CGAATTTTGAGAAGATGATCTGAGTGT
**CXCL9**	Forward primer:	TCGTCCTGGGGAAAACCCTA
	Reverse primer:	GGAAACTGTAGCCACGGTGA
**CCL5**	Forward primer:	CCAGGACTTGGGGAGTTTCC
	Reverse primer:	TGGACTGGAGGGCAGTTAGA
**CCR7**	Forward primer:	CTGGGCCACAAGGTATGTGA
	Reverse primer:	ACCGTTCAACAGACCTCACC
**MMP10**	Forward primer:	CTGTGCTGCTGTCACATACC
	Reverse primer:	ACCCCAGGCTTACAGGACAA
**CCL7**	Forward primer:	GGTGGCAAGAAGTAGGGTGT
	Reverse primer:	TGGTGTCAGCTTGTCAGAGAC
**MMP14**	Forward primer:	GAGGAAACCCTTGGCAAACC
	Reverse primer:	CCACCACTACCCTTCGTGTC
**il1β**	Forward primer:	GGGGAAGAGGCTATTGCTACC
	Reverse primer:	ATGCCCATTTCCACCACGAT
**Fgf1**	Forward primer:	ACCGAGAGGTTCAACCTGCC
	Reverse primer:	GCCATAGTGAGTCCGAGGACC
**CCL2**	Forward primer:	GAGAGCAACACAGGTTGGGA
	Reverse primer:	GGAAGGACTGGGGCTTTTGT
**CXCL2**	Forward primer:	GCTACTAGCTGGAGTCTCCCT
	Reverse primer:	AGCTGTTCCTTGGGGGAAAG
**Edn1**	Forward primer:	TGAAAACCCCCAAGAGGTGAT
	Reverse primer:	CCTTCAAGGTCAACCAGCCA
**igf1**	Forward primer:	GATACACATCATGTCGTCTTCACA
	Reverse primer:	CAGTACATCTCCAGTCTCCTCAGA
**Actin**	Forward primer:	GGTCATCACTATTGGCAACG
	Reverse primer:	ACGGATGTCAACGTCACACT

The quantitative real-time RT-PCR analysis was performed by BioRad SYBR Premix. The reaction mixtures containing SYBR Green were composed following the manufacturer's protocol. Relative expression levels of the target genes were normalized with the control gene Actin.

### Conditioned medium preparation

Macrophage polarization was obtained by culturing cells in DMEM medium supplemented with 10% FBS and 10 ng/mL IL-13 or 1 mM metformin for three days. Then different polarized RAW264.7 cells were incubated in serum free medium for 24 h, after which culture supernatants were collected as conditioned medium (CM). CM was centrifuged at 3000 rpm to separate out the debris and stored at −80°C.

### Wound healing assay

Mouse endothelial cells were seeded in 24-well plates and cultured until 70–80% confluent. A straight scratch was made by using a pipette tip, formatting an artificial wound. Cells were incubated with conditioned medium for 12 h. To assess the migration of cells across this artificial wound, five optical fields (10 × magnification) were randomly chosen, analyzed by using a LEICA DMI 4000B microscope with Leica Application Suite software.

### Tube formation assay

The MMVECs (2 × 10^4^ cells per well) were seeded in 96-well plates which have been filled with 50 μl matrigel and solidified in 37°C. Cells were cultured in CM supplied with 2.5% FBS for 6 hours. To observe the formation of tube-like structures, five optical fields (10× magnification) per well were randomly chosen and analyzed by a LEICA DMI 4000B microscope with Leica Application Suite software.

### Trans-well assay

A Trans-well Boyden Chamber (Costar, Bethesda, MD, USA) was used for migration assay. LLC cells or MMVECs were seeded in a density of 1 × 10^4^ (in 200 μl CM) per well in the upper chamber. The lower compartment contained 0.6 mL CM. After 24-hour incubation at 37°C, the cells were fixed with 90% EtOH for at least 30 min, and then all of the non-migrant cells were removed from the upper chamber with cotton buds dipped in PBS and discarded. The migrated cells on the bottom part of the filter stained with 0.1% crystal violet. The stained cells were subsequently photographed by a LEICA DMI 4000B microscope. For the analysis, five optical fields (10 × magnification) per well were randomly chosen and quantitative analyzed by Image J software.

### Western blot analysis

After treatment with compounds for the indicated times, the macrophages were harvested. Then, cellular and nuclear extracts were prepared and analyzed as previously described [28].

### Statistical analyses

Values are presented as mean ± SD. Two-tailed and unpaired Student's *t* test was used for statistical analysis, and differences were considered significant for *p* values less than 0.05.

## SUPPLEMENTARY FIGURES


