# Supplementary material for: Metformin prevents cancer metastasis by inhibiting M2-like polarization of tumor associated macrophages
Source: Oncotarget. 2015 Oct 19;6(34):36441–55. doi: 10.18632/oncotarget.5541 (PMC4742188; doi:10.18632/oncotarget.5541)
Supplement: Supplementary file 1 [file oncotarget-06-36441-s001.pdf]

SUPPLEMENTARY FIGURES

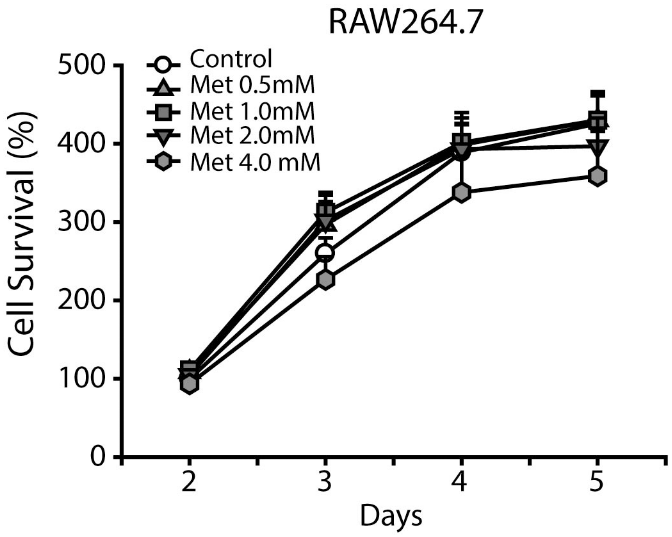

Supplementary Figure S1: The impact of metformin on RAW264.7 proliferation. Cells were seeded in 96-well plates and exposed to serial concentrations of metformin for 5 days. SRB assays were used to examine the cell-proliferation inhibitory activities of metformin. Each condition was performed in triplicate independently, and the error bars represent the standard deviation.

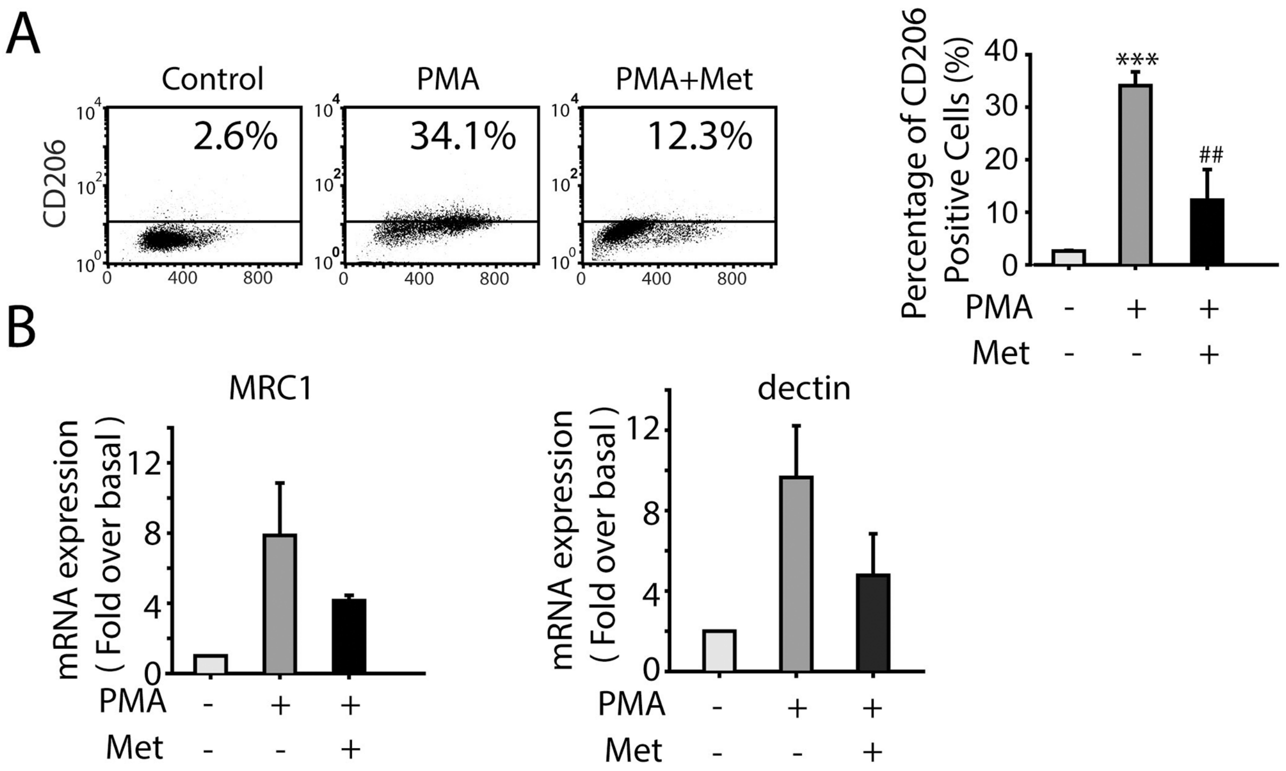

Supplementary Figure S2: Metformin inhibits M2-like polarization of THP-1 induced by PMA. A. THP1 were treated with PMA(10 ng/ml) or the combination with metformin(1 mM) for 48 h and expression of M2 marker CD206 were determined by FACS analysis. **B.** mRNA expression of M2 marker gene (MRC1 and dectin) was analyzed by quantitative RT-PCR. The histogram bars represent three biological replicates, displayed as mean ± SD. ***$p < 0.001$ for PMA versus Control. ##$p < 0.01$ for PMA+Met versus PMA.

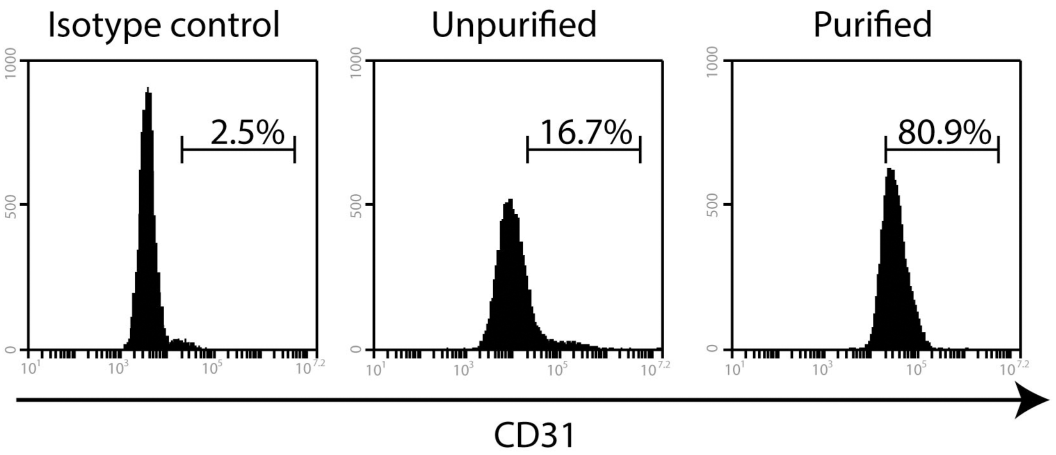

Supplementary Figure S3: The characterization of MMVECs purified from murine lung. Both primary cells and purified cells isolated were stained with the anti-CD31 antibody and the percentage of $CD31^+$ cells were analyzed by FACS analysis.

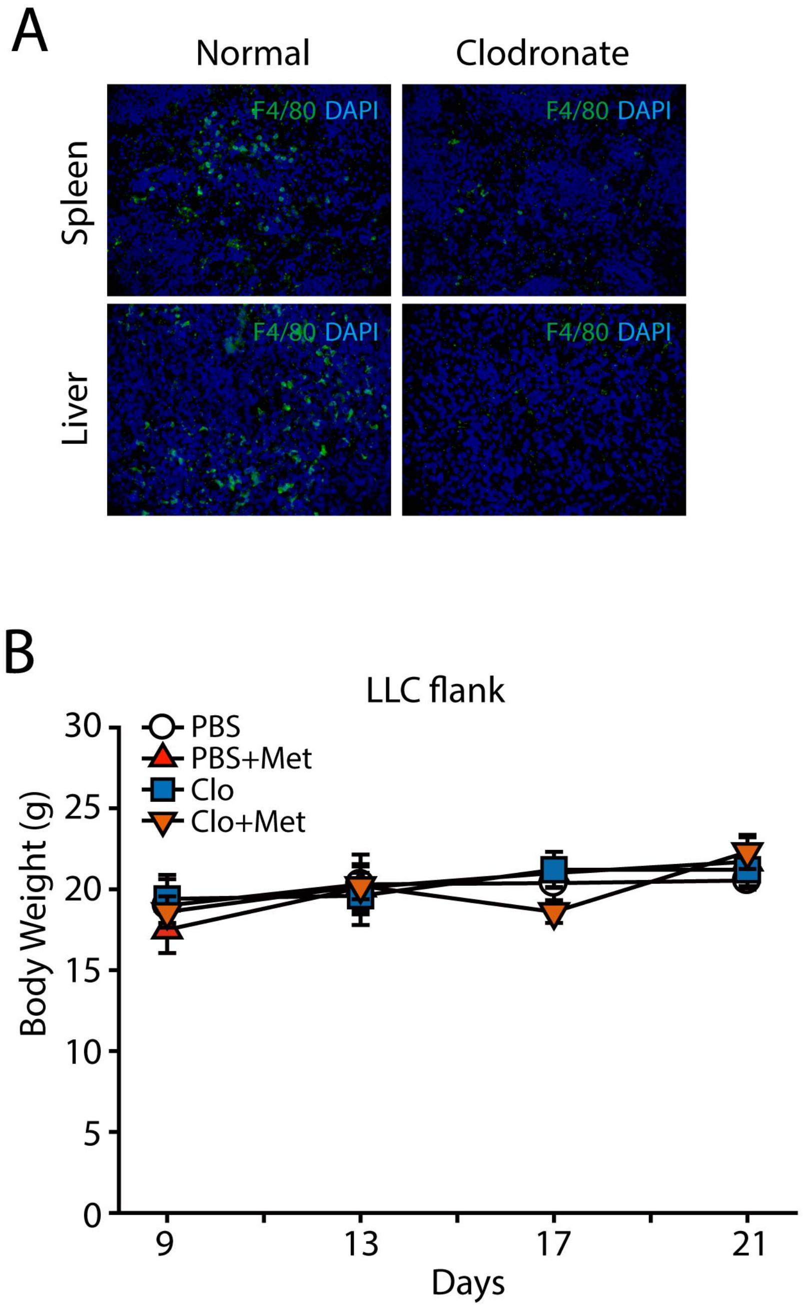

Supplementary Figure S4: A. Macrophages were depleted by clodronate liposome injection *in vivo***.** To evaluate the efficiency of macrophage depletion, both liver and spleen tissues were stained for F4/80. **B.** The average body weight of each group is expressed as the mean ± SD, $n = 8$.

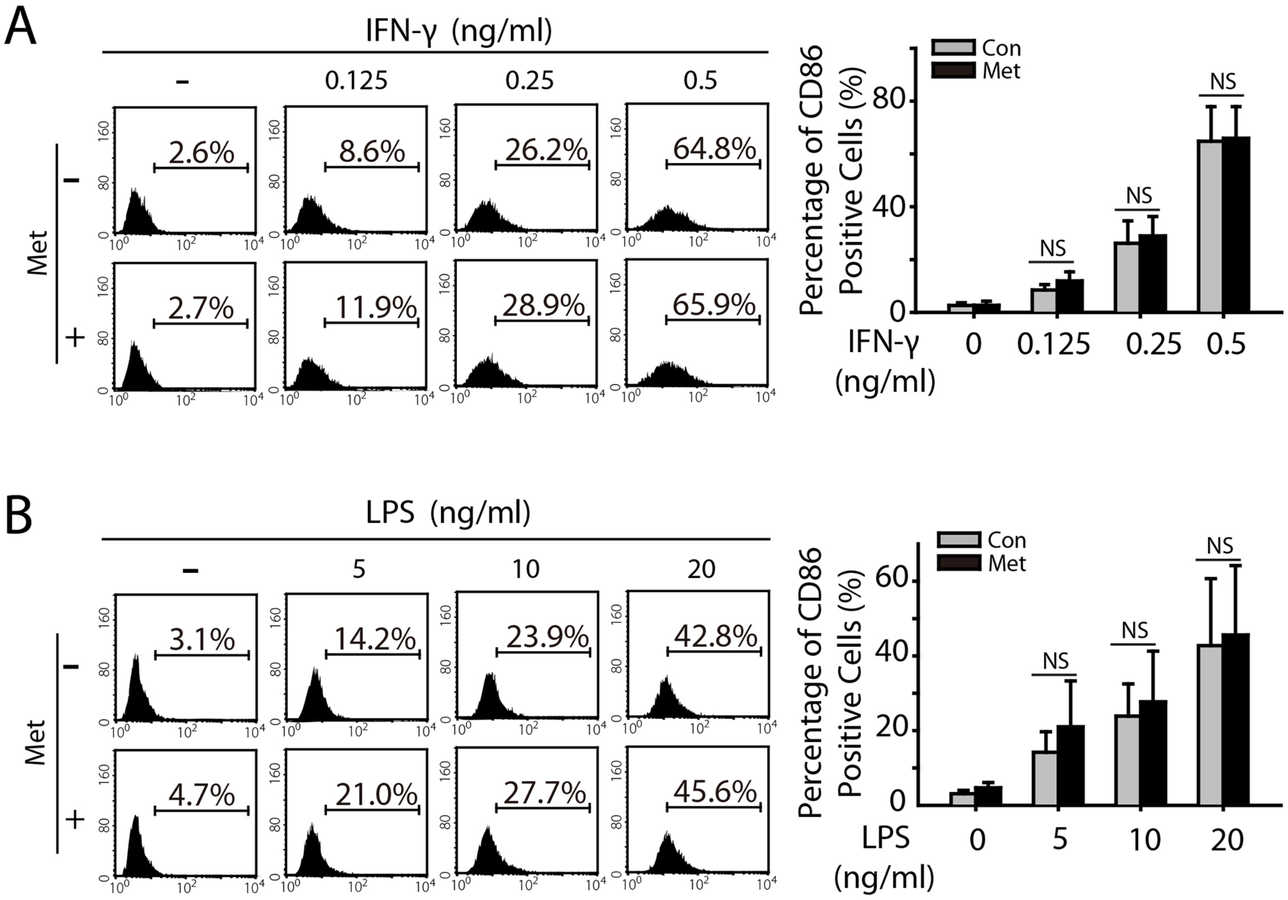

Supplementary Figure S5: Metformin doesn't inhibit M1-like polarization of macrophages induced by IFN-γ and LPS. **A.** RAW264.7 were treated with metformin (1 mM) and different concentrations of IFN-γ or the combination for 48 h. The expression of M1 marker CD86 was analyzed by FACS analysis. **B.** RAW264.7 were treated with metformin (1 mM) and different concentrations of LPS as indicated and the expression of M1 marker CD86 were analyzed by FACS analysis. The histogram bars represent three independent experiments. NS means no significance as evaluated using Student's *t* test.

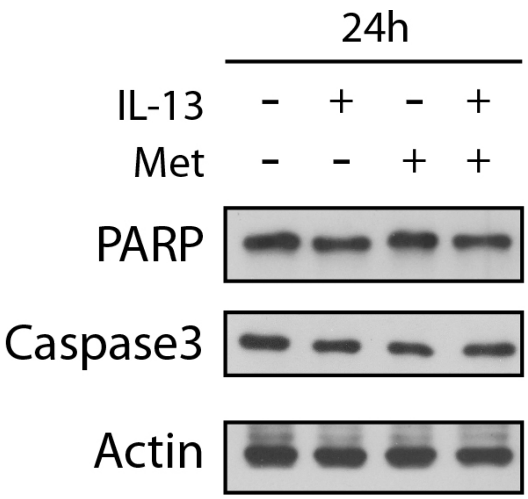

Supplementary Figure S6: To exclude the impact of CM on tumor cell survival, LLC cells were treated with the conditioned medium for 24 h and caspase3 activation and PARP cleavage were analyzed by Western blot. No significant difference was found in four groups.
